# Supplementary material for: The interactome of intact mitochondria by cross-linking mass spectrometry provides evidence for coexisting respiratory supercomplexes
Source: Mol Cell Proteomics. 2017 Dec 8;17(2):216–32. doi: 10.1074/mcp.RA117.000470 (PMC5795388; doi:10.1074/mcp.RA117.000470)
Supplement: Supplemental Data [file supp_RA117.000470_133922_0_supp_23978_fzffwf.zip › spectra_annotation/mito_spectra_annotation/215-1-4-1-7-1.pdf]

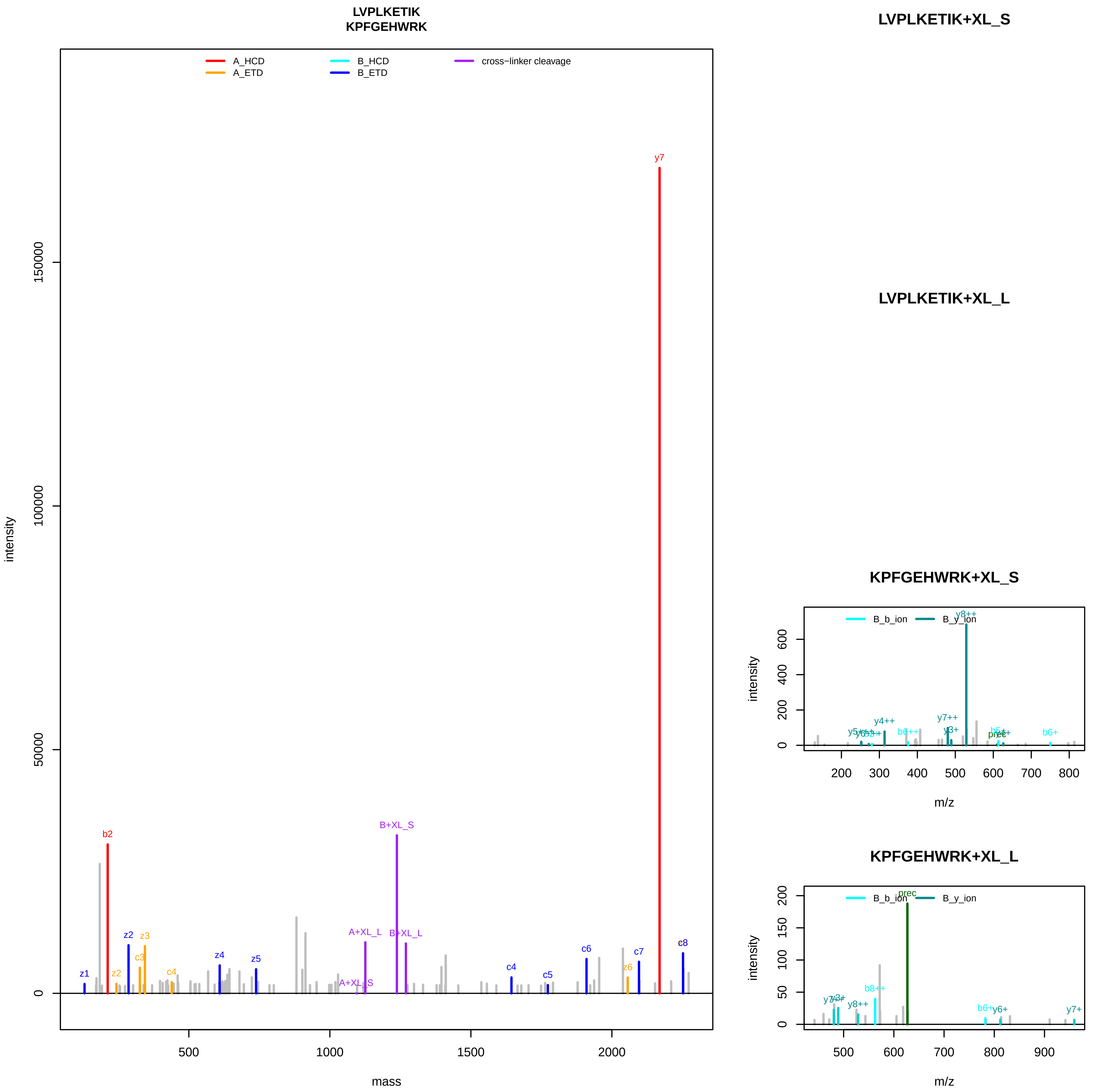
LVPLKETIK
KPFGEHWRK
A_HCD
A_ETD
B_HCD
B_ETD
cross−linker cleavage
intensity
mass
y7
b2
B+XL_S
A+XL_L
B+XL_L
A+XL_S
z1
z2
z3
c3
z4
c4
z5
c4
c5
c6
z6
c7
c8
LVPLKETIK+XL_S
LVPLKETIK+XL_L
KPFGEHWRK+XL_S
B_b_ion
B_y_ion
y8++
y7++
y4++
prec
b6+
m/z
KPFGEHWRK+XL_L
b8++
y8++
y6+
y7+
